# Processing of unconventional stimuli requires the recruitment of the non-specialized hemisphere

**DOI:** 10.3389/fnhum.2015.00032

**Published:** 2015-02-09

**Authors:** Yoed N. Kenett, David Anaki, Miriam Faust

**Affiliations:** ^1^The Leslie and Susan Gonda (Goldschmied) Multidisciplinary Brain Research Center, Bar-Ilan UniversityRamat-Gan, Israel; ^2^Department of Psychology, Bar-Ilan UniversityRamat-Gan, Israel

**Keywords:** creativity, expertize, un/conventional processing, hemispheric specialization, non-specialized system, Mooney faces

## Abstract

In the present study we investigate hemispheric processing of conventional and unconventional visual stimuli in the context of visual and verbal creative ability. In Experiment 1, we studied two unconventional visual recognition tasks—Mooney face and objects’ silhouette recognition—and found a significant relationship between measures of verbal creativity and unconventional face recognition. In Experiment 2 we used the split visual field (SVF) paradigm to investigate hemispheric processing of conventional and unconventional faces and its relation to verbal and visual characteristics of creativity. Results showed that while conventional faces were better processed by the specialized right hemisphere (RH), unconventional faces were better processed by the non-specialized left hemisphere (LH). In addition, only unconventional face processing by the non-specialized LH was related to verbal and visual measures of creative ability. Our findings demonstrate the role of the non-specialized hemisphere in processing unconventional stimuli and how it relates to creativity.

## Introduction

Creativity has been considered an elusive concept, being both theoretically and empirically difficult to investigate (Abraham, 2013). It is multi-faceted, and specific tasks measure only partial aspects of this complex concept (Kaufman et al., 2012; Runco and Jaeger, 2012). Nevertheless, in recent years a new neurocognitive view on creativity has emerged (Dietrich, 2004; Dietrich and Kanso, 2010; Jung et al., 2010, 2013; Abraham, 2013). One finding that is consistently observed in this research is the strong involvement of the right hemisphere (RH) in creative processes (Arden et al., 2010; Dietrich and Kanso, 2010; Mihov et al., 2010; Gonen-Yaacovi et al., 2013). In fact, some view the RH as the “seat” of creativity, while others regard the genesis of creativity as resulting from the interactions of neural networks in the two cerebral hemispheres (Lindell, 2010; Gonen-Yaacovi et al., 2013; Jung et al., 2013).

The unresolved role of the RH in the creative process is mainly a result of the extensive research on hemispheric involvement in language processing (Faust, 2012). While the left hemisphere (LH) has been shown to be the specialized system for conventional language processing, evidence indicates that the RH plays a unique role in processing more unconventional, creative facets of language (Faust, 2012). This has been shown in linguistic ambiguity resolution and novel metaphor comprehension, which both require processing unusual and seemingly unrelated semantic relations (Faust and Chiarello, 1998; Chiarello, 2003; Mashal et al., 2005; Lindell, 2010; Mihov et al., 2010; Gold et al., 2011; Faust, 2012; Mirous and Beeman, 2012). For example, Pobric et al. (2007) used repetitive transcranial magnetic stimulation (rTMS) to study hemispheric processing of novel metaphoric expressions. They found that while RH interference disrupted novel metaphor expression processing, LH interference disrupted regular, conventional expression processing, but facilitated novel metaphor expression processing. Thus, research suggests that processing of semantic creativity is facilitated by the involvement of the opposite, non-specialized hemisphere (Jung-Beeman, 2005; Mirous and Beeman, 2012). This view raises the question of whether an opposite pattern of hemispheric involvement for RH-specialized domains will emerge. Specifically, will the non-specialized LH be more involved in unconventional processing in RH-specialized realms? The present paper addresses this issue.

Recently, Aziz-Zadeh et al. (2013) investigated RH involvement in creativity. The authors conducted a fMRI study where participants performed both a visual creative and a visual control task. In the creative, divergent thinking, task participants were required to manipulate three shapes (e.g., “c”, “0”, “8”) and create a recognizable object (e.g., a smiley face). In the control, convergent thinking, task participants were required to mentally rotate three parts of a trisected shape in order to reconstruct the original shape (e.g., rectangle). Unexpectedly, they found that the creative task recruited the LH more strongly than the control task (left posterior parietal cortex, motor areas and dorsolateral prefrontal cortex). The control task, however, more strongly recruited the RH compared to the creative task (right posterior parietal cortex, precuneus; see Aziz-Zadeh et al., 2013 for a complete description). The authors noted that their findings underscore the importance of the activation of the non-specialized hemispheric system in visual divergent thinking. Thus, the findings of Aziz-Zadeh et al. provide neural evidence that creativity in the visual domain depends on the increased activation of the non-specialized LH.

Based on these latter findings, combined with evidence from linguistic creativity research (Faust, 2012), we propose a novel neurocognitive hypothesis for the creative process. We suggest that once a stimulus is presented, the specialized hemisphere for that stimulus is responsible for its processing. However, when the stimulus is unconventional, the specialized hemisphere cannot efficiently process the stimulus by itself and the non-specialized hemisphere is recruited. In the present study, we investigate our proposal in a task that is considered to be strongly RH specialized, namely face perception (Yovel et al., 2008).

The strong involvement of the RH in face perception has been long established. In healthy individuals, the superiority of the RH in face recognition has been shown, in both behavioral and neuroimaging studies, to be a stable individual characteristic (Luh, 1991; Yovel et al., 2008). For example, faces presented to the left visual field, directly projected to the RH, are processed faster and more accurately than faces presented to the right visual field (e.g., Yovel et al., 2008; Dien, 2009). This has also been consistently shown in brain-injured patients. It is widely agreed that a RH lesion is necessary to induce face perception deficits (prosopagnosia), and some even claim that it is also sufficient (for a review, see Mayer and Rossion, 2007).

Face processing is considered to be based upon two parallel types of processing—featural and holistic-configural (e.g., Renzi et al., 2013). Featural processing refers to the analysis of single components in a face (such as nose, eyes, etc.). Configural processing, on which human expertize in face perception heavily depends upon, computes the spatial metric relations between the features (i.e., the distance between the mouth and the nose) and integrates it into a unified, gestalt-like representation of the face (Maurer et al., 2002). Studies investigating the contribution of featural and configural processing to face recognition have shown that they involve separate brain regions: While featural processing is considered to be a LH dominant process, configural processing is considered a RH dominant process. This has been shown in various techniques, such as fMRI, positron emission tomography (PET) and electroencephalography (EEG; Rossion et al., 2000; Scott and Nelson, 2006; Lobmaier et al., 2008). For example, in a PET study conducted by Rossion et al. (2000), hemispheric differences were found when participants attended either featural or configural aspects. When participants were required to attend to featural facial aspects, the result was reduced activation in the RH face-selective brain area (fusiform gyrus) and enhanced LH homologous region activation. In contrast, when participants were required to attend to configural face aspects, a RH face selective brain area advantage appeared. Moreover, studies on patients with either unilateral right or left tempero-parietal junction lesions found an asymmetry in local and global featural processing (which is equivalent to featural and relational processing, respectively; Kimchi, 1992; Behrmann et al., 2006): While patients with RH lesions showed better local (featural) processing, LH lesioned patients showed better global (configural) processing (Robertson et al., 1988). Recently this asymmetry was also shown in transcranial magnetic stimulation (TMS) research, thus establishing a more causal relationship between hemisphere and processing strategy (Renzi et al., 2013).

**Figure 1:**
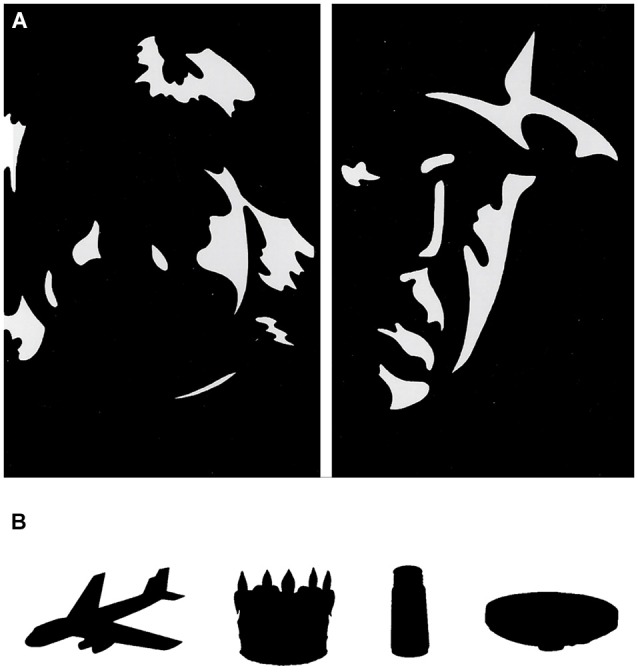
**Examples of stimuli used in the experiments. (A)** Mooney Faces stimuli used in the experiment, varying in sex, age, and pose. Stimuli taken from Latinus and Taylor (2005). **(B)** Examples of the silhouettes chosen for each difficulty category, from left to right: easy—airplane; medium—crown; hard—saltshaker; very hard—belt. Silhouettes created by Wagemans et al. (2008) from the original Snodgrass and Vanderwart (1980) stimuli.

To examine our proposed hypothesis in face processing, we must investigate hemispheric processing of conventional and unconventional face stimuli and examine the relation of these processes to facets of creative ability. However, it is yet to be determined which face stimuli can be considered unconventional faces (similar to linguistic unconventional stimuli). Unconventional faces must consist of mainly featural properties and lack configural properties, thus eliminating RH advantage for processing such faces. Possible face stimuli that may be considered unconventional are Mooney faces (Mooney, 1956). Mooney faces are two-tone impoverished images of faces, in which key diagnostic features, that define usual face configuration, are obscured (i.e., Figure 1A). Indeed, findings show that Mooney faces involve different perceptual processing than natural face processing, as evident in differential electrophysiological patterns (Latinus and Taylor, 2005, 2006). While natural faces elicit greater posterior activation in the RH, Mooney faces elicit greater activation in the LH (Latinus and Taylor, 2006). It has also been shown that individuals with prosopagnosia, usually associated with unilateral RH lesions, exhibit normal Mooney face recognition (Busigny et al., 2010; see Steeves et al., 2006 for a possible double dissociation). Therefore, Mooney faces can be considered unconventional face stimuli. To date, the relationship between Mooney face processing and characteristics of creative ability has not been examined.

In the current study we first investigated, through the use of Mooney faces, whether unconventional visual recognition is related to aspects of creative ability (Experiment 1). After establishing this association we examined whether unconventional visual recognition (i.e., Mooney faces) is processed better by the non-specialized system (i.e., LH) and the relation of this processing to creative ability (Experiment 2).

## Experiment 1

In Experiment 1 we examined whether the processing of unconventional visual stimuli, such as Mooney faces, is related to creative ability. This was done by examining the relationship between Mooney faces identification, and verbal and visual aspects of creative ability. To assess the latter we used tasks that measure divergent thinking, which estimate the potential for creative performance (Runco and Acar, 2012). As a control task, we investigated the relationship between recognition of silhouetted objects’ and the divergent creativity tasks. In contrast to the lateralized nature of face perception, object recognition is considered to activate wide bilateral occipito-temporal and frontal areas (Pennick and Kana, 2012). We, therefore, hypothesized that while Mooney face perception will be related to divergent aspects of creativity, silhouette recognition will not.

### Method

#### Participants

Twenty eight participants (20 males, 8 females; mean age 27.79 [SD = 5]) took part in Experiment 1. All participants had normal or corrected to normal vision and reported that they were right-handed. Participants either volunteered for the experiment or received course credit. The experiment was approved by the Bar-Ilan University institutional review board.

#### Ambiguous visual processing tasks

##### Mooney face identification

60 Mooney faces, varying in sex, pose direction and age, were selected. Participants were required to detect the face as quickly as possible. Once detected, the stimuli disappeared and participants had to identify three of its features: sex (male/female), pose direction (up, down, left, right, and center) and approximate age (young, adult, old). For each of these questions, only one correct answer was accepted, predetermined by two independent judges (Yoed N. Kenett (YNK) and David Anaki (DA), inter-rater agreement of *r* = 0.92).

##### Silhouettes identification task

The task consisted of a silhouette version of the Snodgrass and Vanderwart (1980) visual stimuli (created by Wagemans et al., 2008). Two independent judges (YNK and DA) judged the recognition difficulty of 260 silhouettes on a four-point scale (easy, medium, hard, and very hard), with an inter-rater agreement of *r* = 0.9. Fifteen silhouettes were selected for each category, resulting in 60 silhouettes (Figure 1B). In this task, participants were presented with the stimuli and were required to recognize them as quickly as possible. Once recognized, the stimuli disappeared and the participants were instructed to write the exact identity of the object they saw.

#### Divergent thinking tasks

In order to measure participants’ divergent thinking, we used a standard battery of divergent thinking tests (see Kaufman et al., 2012 for a current review of creativity measurements). Divergent thinking is considered the hallmark predictor of creative ability characteristics (Runco and Acar, 2012), and is frequently used in creativity research (i.e., Baird et al., 2012).

In a divergent thinking task, participants generate responses to verbal or figural cues (Runco and Acar, 2012). A few examples of various divergent thinking tasks are the alternative uses task (i.e., what are the possible uses of a brick?), similarities between concepts (i.e., how are carrot and potato similar?) or line meaning (i.e., given an abstract line drawing, what are all the possible meanings of this drawing?; Wallach and Kogan, 1965; Torrance, 1966; Silvia et al., 2008). Divergent thinking is considered a valid and reliable measure of creative ability (Runco and Jaeger, 2012). Two key measures of divergent thinking tasks are fluency and subjective quality of responses. The fluency measure reflects the amount of responses generated by a participant to a given stimuli (Runco and Acar, 2012). The quality measure reflects various creative features of a participant’s response. The estimation of quality is achieved by independent judges, who evaluate various creative features of the response, such as novelty and unusualness (Silvia et al., 2008, 2013; Runco and Jaeger, 2012). In the present research, we measured quantitative and qualitative divergent thinking, with the Tel-Aviv Creativity Test (TACT; Milgram and Milgram, 1976).

##### Tel-Aviv University Creativity Test (TACT; Milgram and Milgram, 1976)

This test is a modified Hebrew version of the Wallach and Kogan (1965) battery of creativity tests. The test is comprised of four sub-tests—two verbal (alternative uses and pattern matching tasks), and two visual (similarities and line meanings tasks). Each sub-test lasts six minutes and is comprised of four open questions. The results of the verbal and visual sub-tests of the TACT were combined into TACT verbal and TACT visual scores. For each sub-test two scores were produced—fluency (number of responses provided) and quality (originality and applicability of response; Jauk et al., 2013). The fluency score was calculated by counting the number of different answers supplied by the participant. The quality score was determined by three independent judges who evaluated the originality and applicability of the infrequent responses, namely, answers which appeared in 5% or less of the sample (Milgram and Milgram, 1976). In the final stage, the originality and applicability scores of each answer were transformed into a 1–10 scale, which determined the quality of the answer. Only answers with a qualitative score higher than three were accepted. Thus, the quality score of a specific participant to a specific item in the TACT is the amount of qualitative answers (scoring more than three on the quality scale) which were generated. Inter-rater agreement between judges on the subjective judgments of the quality answers was 0.65.

#### Procedure

Each participant performed the three tasks (Mooney faces, silhouettes, TACT) in a random order. The visual tasks were conducted using the E-prime software (Schneider et al., 2002). The stimuli (12.4° wide × 15.6° long) were presented centrally, against a black background, on a standard CRT computer screen. Participants were instructed to recognize the stimuli as fast as they could by pressing the spacebar key. Once they pressed the key, the stimuli disappeared and they had to answer the different questions in the two tasks: For the Mooney face task, the participants had to press a number key—two options for the sex recognition task (1 - male, 2 - female), five options for the pose direction recognition (1 - up, 2 - down, 3 - left, 4 - right, and 5 - center), and three options for the approximate age recognition (1 - young, 2 - adult, 3 - old). For each question, a slide appeared with the various options for that specific question, prompting the participant to make a choice. Participants were free to use whichever hand they preferred to make their decisions. Once the participant completed a trial, s/he was immediately presented with the next trial. For the silhouette task, the participants typed their answers, which were recorded by the E-Prime software and later manually analyzed. Both visual tasks included a short four-trial practice consisting of stimuli that were not used in the task itself. Participants were instructed to respond as quickly and accurately as they could. For the TACT task, participants were informed that they would undergo a test that measures creative ability.

#### Analysis

For the two visual tasks we analyzed only the participant’s accuracy due to the very slow reaction times (RT) of these tasks. For the Mooney face recognition task the average RT was 2500 ms and for the silhouettes recognition task the average RT was greater than 1500 ms. These long RTs did not allow sensitive analysis of performance in the two visual tasks. Each visual condition was examined for conditions of application. This was done by conducting a Kolmogorov-Smirnov test of normality and by examining the skewness and kurtosis. Finally, Fleiss Kappa (Fleiss, 1971; de Mast, 2007) was used to examine the internal consistency of each of the visual conditions. We found our data to be skewed in most cases (see below), yet we conducted ANOVA analysis as it is generally robust against the normality assumption (Schmider et al., 2010), as long as the homogeneity of variance assumption is kept (Glass et al., 1972). A one-way repeated-measures analysis of variance (ANOVA) was conducted to investigate the average accuracy for the different questions of the Mooney identification task (sex, pose, age) and the difficulty levels of the silhouette task (easy, medium, hard, and very hard). A Greenhouse-Geisser correction was used to correct for violation of sphericity. *Post hoc* analyses were conducted via a Tukey honest significant difference (HSD). Finally, we conducted *post hoc* analyses to examine the power of the two visual tasks (Onwuegbuzie and Leech, 2004; Button et al., 2013), with the G*power program (Faul et al., 2007).

To examine the relationship between the visual tasks and the TACT verbal and visual scores, we conducted a correlation analysis between the visual tasks and the TACT scores, with a 2-tailed Pearson correlation. To control for family-wise error rate (Rosenblatt, 2013), we performed the correlation analysis on six variables only: accuracy rates of the Mooney faces sex recognition (Mooney_s), accuracy rates of the silhouette easy category (Sil_easy), TACT verbal quality score (Verb_Q), TACT verbal fluency scores (Verb_F), TACT visual quality scores (Vis_Q) and TACT visual fluency scores (Vis_F). These specific visual tasks were chosen as they resulted in the highest accuracy ratings (see below), thus allowing us to optimally examine the relation between these visual tasks and the TACT scores. Finally, we conducted a *post hoc* power analysis to examine the power of each of the significant correlations using the G*power program (Onwuegbuzie and Leech, 2004; Faul et al., 2007; Button et al., 2013).

### Results

**Figure 2:**
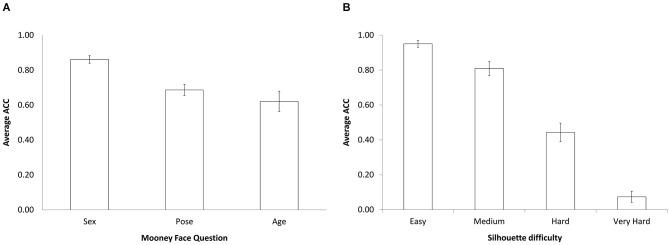
**Behavioral analysis of the two visual tasks. (A)** Mooney face accuracy analysis—X-axis represents the three questions of the task, Y-axis—mean accuracy rate (including error bars). **(B)** silhouette accuracy analysis—X-axis represents the four difficulty categories of the task, Y-axis—mean accuracy rate (including error bars).

Analysis of the Mooney face recognition task revealed differences in accuracy for the three questions (sex, pose, and age), *F*_(1,34)_ = 38.77, *p* < 0.001, *η*^2^ = 0.589 (Figure 2A). *Post hoc* analysis showed greater accuracy in the sex identification task than in the pose and age identification tasks (all *p*’s < 0.001). Power analysis of the Mooney task indicated that with our sample size and effect size estimation, we had sufficient power to detect a significant main effect (power = 0.99). Analysis of the silhouette task revealed a significant effect of stimuli difficulty on the mean identification accuracy, *F*_(2,64)_ = 487.704, *p* < 0.001, *η*^2^ = 0.948 (Figure 2B). *Post hoc* analyses revealed that each difficulty level significantly differed from all others (all *p*’s < 0.001), with a rapid decline of mean accuracy from 0.95 accuracy rate in the easy category to 0.07 accuracy rate in the very hard category. Power analysis of the silhouette task indicated that with our sample size and effect size estimation, we had sufficient power to detect a significant main effect (power = 0.99).

Analysis of the Mooney face sex recognition task revealed good internal consistency (*k* = 0.72). Furthermore, this condition passed the Kolmogorov-Smirnov test of normality (*p* = 0.2) with skewness of −0.25 (SE = 0.44) and kurtosis of −0.73 (SE = 0.85). Analysis of the silhouette easy category did not pass the Kolmogorov-Smirnov test of normality (*p* < 0.001) with skewness of −0.83 (SE = 0.44) and kurtosis of 0.12 (SE = 0.86). This task had a high inter-rater agreeability (88%).

The correlation analysis between the visual tasks and TACT verbal and visual scores revealed a significant positive correlation between Mooney_s and Verb_F scores (*p* < 0.04). Power analysis of the correlation between Mooney_s and Verb_F indicated that with our sample size and correlation score, we had high power to detect a significant correlation (power = 0.53). Furthermore, this analysis revealed a positive correlation which approached significance between Mooney_s and TACT Verb_Q (*p* = 0.08) (Table 1). Power analysis of the correlation between Mooney_s and Verb_Q indicated that with our sample size and correlation score, we had medium power to detect a significant correlation (power = 0.39).

**Table 1:** **Correlation analysis of accuracy ratings of the visual task conditions and creativity scores**.

Variables	1	2	3	4	5	6
1—Mooney_s	–	−0.12	0.32^†^	0.38*	0.12	0.17
2—Sil_easy		–	−0.26	−0.22	−0.22	−0.25
3—Verb_Q			–	0.88**	0.53**	0.63**
4—Verb_F				–	0.70**	0.79**
5—Vis_Q					–	0.94**
6—Vis_F						–

Note. Mooney_s—Mooney faces sex task; Sil_easy—silhouettes easy category; Verb_Q—Verbal creativity quality scores; Verb_F—Verbal creativity fluency scores; Vis_Q—Visual creativity quality scores; Vis_F—Visual creativity fluency scores; ^†^—p < 0.1; *—p < 0.05; **—p < 0.01.

### Discussion

In Experiment 1, we examined the relationship between processing of unconventional visual tasks and aspects of verbal and visual creative ability. Our hypothesis was that if creative ability is related to processing of unconventional stimuli, as evident in linguistic creativity (Mirous and Beeman, 2012), a relationship between unconventional visual processing and creativity will be found. To date, no research has examined the relationship between creative ability and processing of unconventional visual stimuli. In Experiment 1, two unconventional visual tasks were administered; one which is more typically lateralized to the RH (face recognition) and one which is distributed more bilaterally (object recognition). Furthermore, participants were measured for divergent visual and verbal creative ability, using both quantitative (amount of responses) and qualitative (quality of responses) means. The results show a relationship between verbal creative ability and the recognition of unconventional faces: A positive correlation was found between the Mooney recognition task and the TACT verbal quality and fluency scores. Interestingly, this significant relationship between Mooney face processing and TACT scores was found in the TACT verbal, but not visual, score. Although the correlations in the TACT visual score were similar to those in the TACT verbal score, they did not reach significance. These weak correlations may be due to the small sample size used in Experiment 1. For the silhouette task, no significant correlation with the TACT was found, which might indicate that the silhouette recognition task requires different, non-creative processes than those required for the Mooney face recognition task.

Experiment 1 indicates that unconventional Mooney face recognition is related to facets of creative ability, thus allowing us to explore our proposed hypothesis; while conventional face recognition will be better processed by the specialized RH system, unconventional face recognition, whose processing requires creative capacities, will be better processed by the non-specialized LH system. Furthermore, based on our hypothesis, we predicted a positive relationship between unconventional face processing (i.e., Mooney faces) in the LH, and TACT measures of creative ability. In contrast, no correlation was predicted between conventional face processing in the RH and TACT measures of creative ability.

## Experiment 2

In Experiment 2 we directly examined our hypothesis that processing of unconventional visual stimuli (i.e., Mooney faces) will involve the LH. We applied the split visual field (SVF) paradigm, where stimuli presented in one hemi-field is projected and initially processed in the lateral hemisphere, due to the crossing of the nasal optic fibers in the optic chiasm (Bourne, 2006; Hunter and Brysbaert, 2008). Although the information eventually reaches both cerebral hemispheres regardless of its initial presentation, studies show both accuracy and response latency differences that depend on the field of presentation (Dien, 2009). While this technique has its drawbacks and is less effective in assessing individual laterality effects, research contrasting the SVF paradigm with neuroimaging techniques shows that it can validly assess group-based laterality differences. For example, Hunter and Brysbaert (2008) show that when devising an efficient SVF experiment (one that uses well-controlled stimuli and protocol), the SVF paradigm attained a similar level of precision in assessing laterality as when using fMRI measures.

In the present experiment we added a third visual task of natural face recognition as a control (Yovel et al., 2008). We predicted that while natural faces will be processed better by the specialized RH, Mooney faces will be processed better by the non-specialized LH. Additionally, we expected to find a positive relationship between TACT verbal and visual scores and accuracy of Mooney face recognition, but only when Mooney faces are processed initially by the LH, and not by the RH. Finally, in accordance with our hypothesis, we expected to find a negative or no correlation between TACT verbal and visual scores and natural face recognition, when processed by the specialized RH system.

### Method

#### Participants

Seventy-eight participants were initially recruited for Experiment 2. Twelve participants were removed due to high frequency of eye-movements (cutoff was set on >50% eye movement in any of the conditions), two due to incompliance with instructions, and four participants due to low accuracy rates (cutoff <50% accuracy). Analysis was performed on the remaining 60 participants (30 males; mean age 23.45 [SD = 3.5]). All participants had normal or corrected to normal vision. Participants either volunteered for the experiment or received course credit. All participants were dominantly right-handed, with a mean score of 97.96 on the Edinburgh Handedness Inventory (Oldfield, 1971). The experiment was approved by the Bar-Ilan University institutional review board.

#### Stimuli

The tasks were Mooney faces sex recognition, natural faces sex recognition and silhouettes animate/inanimate categorization. The Mooney faces task consisted of 54 stimuli (similar to those used in Experiment 1), equally divided into male and female faces. Participants were presented with a Mooney face and were required to recognize the sex of that face. The silhouette task consisted of 60 stimuli, equally divided into animate and inanimate silhouettes. The silhouette stimuli used in Experiment 2 were chosen from the easy and medium difficulty categories, as classified in Experiment 1. This was done as there were not enough animate stimuli in the easy category to create equal groups of animate and inanimate silhouettes. In this task, participants were presented with a silhouette and were required to recognize whether it is an animate or an inanimate object. The natural faces task consisted of 60 natural color images of faces, equally divided into male and female natural faces (courtesy of Michael Tarr, www.tarrlab.org). In this task, participants were presented with a natural face and were required to recognize the sex of that natural face.

#### Procedure

Participants sat 50 cm from a CRT screen, resting their head on a chin-rest. They were instructed not to move their head and eyes, and were informed that their left eye was being video-taped for later examination. All visual tasks were conducted using the E-prime software (Schneider et al., 2002). The stimuli were presented against a black background and were about 6.8° wide × 5.7° long. In all three tasks, each trial began with a fixation cross appearing at the center of the screen for 200 ms. Then, the stimulus appeared for 120 ms in either the left or the right side of the screen, with the inside edge of the stimulus presented 1.5° from a central fixation point. After the stimulus disappeared, participants were required to respond by either pressing a green key (e.g., male) or a yellow key (e.g., female). They were instructed to position their left index finger on a green key and their right index finger on a yellow key. To control for possible motor bias, the relation of answer (e.g., male or female) to key (green or yellow) was randomly switched between participants. Once the participant responded by pressing a key, a slide appeared to notify the participant that he could blink. S/he then initiated the next trial by pressing any key. This was done to eliminate as many eye blinks as possible during the presentation of the visual stimuli. Each task was preceded by a short practice, consisting of four stimuli that were not presented in the main task. Inter-trial interval varied, depending on the participant’s response. The order of the visual tasks was random as well as the presentation of the stimuli within each block. Participants were instructed to respond as quickly and accurately as possible. After completing the lateralization task, the TACT was conducted.

#### Analysis

During the visual tasks, each participant’s left eye was recorded via a web-cam installed on the chin-rest and transferred to a computer in an adjacent room. An in-house MatLab (MathWorks, 2007) program was written to separate the participant’s entire video footage into single trial video clips, with a time window of 1000 ms after trial onset. This resulted in 174 video segments for each participant (60 Natural faces + 54 Mooney faces + 60 silhouettes), which were all manually scanned for eye movements. Trials containing eye movements, RTs lower than 250 ms, or trials with incorrect responses were removed. Finally, for each participant, trials which were above or below 2.5 SD for each condition were also deleted from final data analysis.

Each visual task condition was examined for conditions of application. This was done by conducting a Kolmogorov-Smirnov test of normality and by examining the skewness and kurtosis. Finally, Fleiss Kappa (Fleiss, 1971; de Mast, 2007) was used to examine the internal consistency of each of the visual conditions. The behavioral tasks were analyzed with a repeated-measure ANOVA performed on RT and accuracy as a function of the following variables: Task (natural faces, Mooney faces, silhouettes) X Visual Field (left VF [RH], right VF [LH]).

Although RT data was skewed, we conducted an ANOVA analysis as it is generally robust against the normality assumption (Whelan, 2008; Schmider et al., 2010), as long as the homogeneity of variance assumption is kept (Glass et al., 1972). A Greenhouse-Geisser correction was used to correct violation of sphericity. Planned contrast analysis between Mooney face processing and natural face processing was conducted via a Tukey HSD. Finally, we conducted a *post hoc* power analysis to examine the power of each of the visual tasks using the G*power program (Onwuegbuzie and Leech, 2004; Faul et al., 2007; Button et al., 2013).

Similar to Experiment 1, we conducted a Spearman correlation analysis, in order to examine the relationship between the visual tasks and TACT scores. To control for family-wise error rate (Rosenblatt, 2013), we conducted the correlation analysis only on six variables: Natural faces presented to the RH (Face_RH), Mooney faces processed to the LH (Mooney_LH), TACT verbal fluency scores (Verb_F), TACT verbal quality scores (Verb_Q), TACT visual fluency scores (Vis_F) and TACT visual quality scores (Vis_Q). These two visual conditions (Faces_RH and Mooney_LH) were chosen as they were the a-priori conditions of interest. Accuracy ratings were chosen as our main dependent variable in the correlation analysis in order to be consistent with the analysis in Experiment 1. We also conducted a *post hoc* power analysis to examine the power of each of the significant correlations found.

### Results

#### Behavioral analysis

Analyzing the RT distributions of the different conditions in the behavioral tasks revealed that none passed the Kolmogorov-Smirnov test of normality (all *p*’s < 0.001). Faces_RH condition had a skewness of 0.7 (SE = 0.31) and kurtosis of 0.2 (SE = 0.61). Faces_LH condition had a skewness of 1.1 (SE = 0.31) and kurtosis of 0.45 (SE = 0.61). Mooney_RH condition had a skewness of 0.99 (SE = 0.31) and kurtosis of 0.05 (SE = 0.61). Mooney_LH condition had a skewness of 0.8 (SE = 0.31) and kurtosis of −0.01 (SE = 0.61). Sil_RH condition had a skewness of 1.5 (SE = 0.31) and kurtosis of 1.67 (SE = 0.61). Finally, Sil_LH condition had a skewness of 1.77 (SE = 0.31) and kurtosis of 3.5 (SE = 0.61).

**Figure 3:**
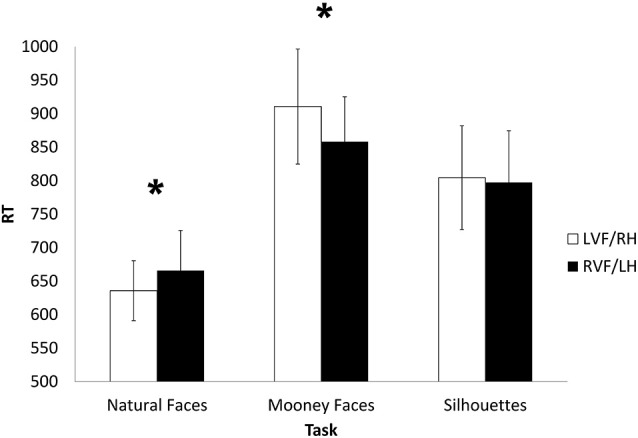
**Mean RT for the different tasks in the right and left visual fields**. Natural Faces—Natural Faces sex recognition task; Mooney Faces—Mooney Faces sex recognition task; Silhouettes—silhouettes in/animate categorization task. LVF/RH—left visual field/right hemisphere; RVF/LH—right visual field/left hemisphere. *—*p* < 0.05.

The behavioral analysis revealed a significant main effect for Task, *F*_(2,107)_ = 21.338, *p* = 0.001, *η*^2^ = 0.266. This effect resulted from faster latency in the natural faces task compared to Mooney faces and silhouettes task (*p*’s < 0.001). No significant difference in RT was found between Mooney faces and silhouettes tasks. Power analysis indicated that with our sample size and effect size estimation, we had high power to detect a significant main effect (power = 0.99). More importantly, a significant interaction was found between Task and Visual Field, *F*_(2,107)_ = 5.114, *p* < 0.01, *η*^2^ = 0.08. This interaction stemmed from differences in the latency of the Mooney faces and natural faces tasks across the two visual fields: while natural faces were processed faster when presented to the left VF, *t*_(118)_ = −1.97, *p* < 0.05, Mooney faces were processed faster when presented to the right VF, *t*_(118)_ = 2.318, *p* < 0.02 (Table 2 and Figure 3). Power analysis indicated that with our sample size and effect size estimation, we had high power to detect a significant interaction effect (power = 0.81). ANOVA analysis of the visual tasks accuracy data revealed a significant main effect of Task, *F*_(2,96)_ = 321.904, *p* < 0.001, *η*^2^ = 0.845. *Post hoc* analyses indicated that this effect was due to a significant difference between the low accuracy of the Mooney faces task compared to the two other tasks (*p*’s < 0.01). No statistical difference was found between the accuracy rates of the natural faces and the silhouette tasks (Table 2). Power analysis indicated that with our sample size and effect size estimation, we had high power to detect a significant main effect (power = 0.99).

**Table 2:** **RT and accuracy of the different tasks in the two visual fields (SD in parentheses)**.

	RT		Accuracy	
	RH	LH	RH	LH
Natural faces	636	666	0.94	0.92
	(117)	(237)	(0.06)	(0.07)
Mooney faces	911	858	0.66	0.68
	(340)	(265)	(0.11)	(0.01)
Silhouettes	804	797	0.93	0.93
	(307)	(305)	(0.07)	(0.07)

*Note. RH—right hemisphere; LH—left hemisphere*.

#### TACT correlation analysis

Analysis of the Mooney_LH condition revealed a low internal consistency (*k* = 0.3). This condition passed the Kolmogorov-Smirnov test of normality (*p* = 0.2) with skewness of −1.71 (SE = 0.31) and kurtosis of 0.13 (SE = 0.61). Analysis of the Faces_RH condition revealed a high internal consistency (*k* = 0.9). This condition did not pass the Kolmogorov-Smirnov test of normality (*p* < 0.001) with skewness of −1.24 (SE = 0.31) and kurtosis of 0.53 (SE = 0.61).

Correlation analysis revealed opposing relations between TACT verbal and visual scores and the visual tasks (Table 3): Mooney faces processed by the LH were positively correlated with Verb_F, Vis_F and Vis_Q (*p* < 0.03 for Verb_F; *p* < 0.01 for Vis_F and Vis_Q). Power analysis indicated that with our sample size and correlation scores, we had high power to detect significant correlations in all comparisons (power = 0.62 for Verb_F, power = 0.82 for Vis_F and power = 0.74 for Vis_Q). This result is compatible with our hypothesis that an unconventional stimulus, processed by its non-specialized system, is related to creative ability. Natural face processing by the RH, on the other hand, was negatively correlated with Verb_F, Vis_F, and Vis_Q (*p* < 0.02 for Verb_F, *p* < 0.05 for Vis_F and *p* = 0.13 for Vis_Q). Power analysis indicated that with our sample size and correlation scores, we had medium power to detect significant correlations for these two comparisons (power = 0.65 for Verb_F and power = 0.49 for Vis_F). According to our predictions this negative relation is expected, as conventional stimuli processed by their specialized system do not require creative processing.

**Table 3:** **Correlation analysis of accuracy ratings of visual task conditions and creativity scores**.

Variables	1	2	3	4	5	6
1—Faces_RH	–	−0.15	−0.30*	−0.14	−0.25*	−0.2
2—M_LH		–	0.29*	0.14	0.36**	0.33**
3—Verb_F			–	0.7**	0.6**	0.64**
4—Verb_Q				–	0.51**	0.58**
5—Vis_F					–	0.88**
4—Vis_Q						–

Note. Faces_RH—natural faces processed by the right hemisphere; M_LH—Mooney faces processed by the left hemisphere; Verb_F—Verbal creativity fluency scores; Verb_Q—Verbal creativity quality scores; Vis_F—Visual creativity fluency scores; Vis_Q—Visual creativity quality scores; *—*p* < 0.05 2-tailed, **—*p* < 0.01 2-tailed.

### Discussion

In Experiment 2, we examined the relationship between processing of unconventional visual stimuli by the two hemispheres and aspects of creative ability. We hypothesized that while conventional faces would be processed better by the RH, unconventional faces would be processed better by the LH. Furthermore, based on our hypothesis, we expected to find a significant positive relationship between processing of unconventional faces by the non-specialized LH and facets of creative ability, as measured by the TACT.

Using a visual field paradigm, participants performed a sex recognition task for conventional (natural) and unconventional (Mooney) faces and an in/animate recognition task of silhouette stimuli. Results showed a significant interaction: natural faces were processed faster by the RH and Mooney faces were processed faster by the LH. Correlation analysis revealed that Mooney faces processed by the LH were positively correlated with TACT scores, whereas natural faces processed by the RH were negatively correlated with TACT scores. Thus, the results of Experiment 2 support our proposed hypothesis regarding the role of the non-specialized system in unconventional processing and creative ability.

## General discussion

In the current study, we propose a novel hypothesis for hemispheric involvement in processing of unconventional stimuli. According to our hypothesis, while conventional stimuli are better processed by the specialized lateralized system, unconventional stimuli are better processed by the non-specialized lateralized system. Thus far, the relationship between a non-specialized system and creative processing has only been shown in the language domain. Specifically, the LH is the specialized hemisphere for processing conventional linguistic stimuli while the non-specialized RH is involved in processing the more creative aspects of language (Faust, 2012; Mirous and Beeman, 2012). However, the question of whether this pattern of specialized/non-specialized lateralized system processing is also relevant to other cognitive domains has not been addressed. In the present study we examined this issue by investigating conventional and unconventional visual face processing, which is a lateralized RH process, and its relationship to creative ability.

In Experiment 1, we investigated whether an unconventional visual task, Mooney face recognition (Mooney, 1956), is related to creative ability, as operationalized by a battery of visual and verbal divergent thinking tests, the TACT (Milgram and Milgram, 1976). The results showed that Mooney face recognition was related to TACT scores, albeit to verbal and not visual tasks. In Experiment 2 we used a SVF paradigm to examine hemispheric processing of conventional (natural) and unconventional (Mooney) face perception, and to investigate the relationship of these conditions to TACT scores. Our results revealed that while natural face recognition was better processed by the RH (as shown by previous studies, Yovel et al., 2008), Mooney face recognition was better processed by the LH. Thus, while both hemispheres processed Mooney faces, the LH exhibited superior processing of such faces compared to the RH. Moreover, correlation analysis revealed that while Mooney faces processed by the LH were positively correlated with TACT scores, natural faces processed by the RH were negatively correlated with TACT scores.

Studying the neural processing involved in a visual creative task, Aziz-Zadeh et al. (2013) found more brain activation in the LH for the creative task (compared to a non-creative task). This research provided initial evidence for the involvement of the non-specialized LH in creative ability. However, the authors only measured neural activation in a creative vs. control visual task, and not the creative ability of their participants. In the work presented here we have gone a step further by directly relating unconventional visual face recognition to individuals’ creative capacities.

What might be the cause for the division between a specialized, conventional processing system and a non-specialized, unconventional processing system? While additional work is required to further elucidate and generalize the proposed hypothesis into other cognitive domains, the answer may lie in the fine-coarse distinction proposed for semantic processing in the language domain (Jung-Beeman, 2005; Mirous and Beeman, 2012). According to this hypothesis the LH specialized system activates fine, conventional semantic meanings, while the RH non-specialized system activates a coarse, unconventional range of meanings, including remote and unusual associations. It is through the interaction of these two systems that optimal semantic processing is achieved. The weaker activation of a wide range of semantic meanings by the non-specialized RH facilitates novel semantic combinations, giving rise to creative products.

In face processing, however, this division seems to be reversed. We suggest that while the RH configural processing of faces allows for unique differentiation and recognition of faces (Renzi et al., 2013), the LH featural processing of faces allows for coarse identification of faces. Similar to the linguistic domain (Jung-Beeman, 2005), the parallel, interactive relationship between these two systems allows for optimal face recognition and may also contribute to creative visual products (Verosky and Turk-Browne, 2012). Unconventional faces such as Mooney faces mainly require featural, and not configural, processing. This is due to the degraded, two-tone, black and white nature of the Mooney face stimuli, which conveys very little configural information, with only approximate identification of global facial structure and features. Therefore, while face recognition is a RH specialized process, unconventional faces such as Mooney faces may be better processed by the non-specialized LH system (see also Cooper and Wojan, 2000).

Finally, what is the underlying factor of the relationship found between non-specialized system processing and facets of creative ability? A specialized, expert system is defined by its efficiency, accuracy and automaticity, which allows efficient processing under conventional conditions (Johnson, 1983). However, this expert system may face difficulties when required to create new, unusual combinations, when demanded to process novel, unconventional stimuli. The involvement of a more flexible, non-specialized system may facilitate this unconventional processing, giving rise to the creative product. Another example of the relationship between non-specialized system and creative ability can be found in the extensive research of Wiley et al. on expertise and creative ability (Wiley, 1998; Wiley and Jolly, 2003). In a series of experiments, Wiley investigated the effects of domain knowledge in creative problem solving. Her research showed that experts tend to get fixated on conventional solutions and are less efficient in finding unconventional solutions, as compared to novices (Wiley, 1998). Wiley and Jolly (2003) also investigated the effects of expert-novice collaboration in creative problem solving tasks. The authors showed that when experts solved problems with novices (or less knowledgeable persons), they increased their solution rates compared to when they work separately (expert-expert and novice-novice). The authors demonstrate how collaboration between a fixated, high knowledgeable expert and a flexible, less-knowledgeable novice brings about efficient creative problem solving. These findings may be generalized to expert vs. non-expert cognitive and neural mechanisms. Thus, creative processing may be highly supported by the interaction between expert specialized and non-expert non-specialized neural networks.

Our hypothesis and findings are supported by the growing amount of research showing the importance of hemispheric communication for creativity (Razumnikova, 2007; Takeuchi et al., 2010; Gonen-Yaacovi et al., 2013; Jung et al., 2013; Yoruk and Runco, 2014; Zhao et al., 2014). For example, Takeuchi et al. (2010) found a significant positive correlation between the size of the corpus callosum and creative ability, which supports the idea that creativity is associated with efficient integration of information through white matter pathways. In a follow up study, these authors conducted a resting state functional imaging research to investigate gray and white matter correlation with intelligence and creativity (Takeuchi et al., 2011). They found a positive relationship between white matter and creativity, further demonstrating the importance of white matter connectivity and creative ability. Fink et al. (2014) examined the relation between gray matter density (assessed via voxel-based morphometry) and various facets of verbal creativity. They found that gray matter density in RH parietal and occipital brain regions was positively correlated with measures of creative ability. Recently, Zhao et al. (2014) conducted a functional connectivity study to examine hemispheric activation in verbal creativity. The authors reported bilateral neural pathway activation, with greater functional connectivity in the RH. This intra-hemispheric activation may be required for the complex interplay between specialized and non-specialized systems in processing conventional and unconventional stimuli. These neurocognitive findings converge with a recent novel theory we have proposed in regard to semantic processing (Faust and Kenett, 2014): We propose that efficient semantic processing is achieved via a balance between rigid, structured semantic processing (as expressed by the specialized linguistic LH lateralized system) and more chaotic, flexible semantic processing (as expressed by the non-specialized linguistic RH lateralized system). Thus, a well-balanced interaction between specialized and non-specialized neurocognitive systems is seemingly critical for the efficient processing of all types of stimuli, and for coping with the less conventional, creative features of reality.

A possible alternative explanation of our results is the hemispheric division of labor account (Banich, 1998a,b; Passarotti et al., 2002; Yoshizaki et al., 2007; Helton et al., 2010). According to this theory, when a lateralized process (i.e., face processing) places unequal resource demands on its specialized hemispheric system (i.e., the RH), it transfers this processing to the other hemisphere (i.e., the LH). This transfer of information facilitates the processing of computationally complex tasks, allowing “shared labor” of the two hemispheres in the required processing (Banich, 1998a). In regard to the results presented here, the division of labor account can provide an alternative explanation in the following way: Mooney faces are processed as regular faces by the RH, but since they are more difficult to process, the RH recruits the LH to assist in processing these faces. However, we believe that this alternative explanation does not hold for the following reasons: First, several studies have shown that Mooney faces are, in fact, processed differently than natural faces (Latinus and Taylor, 2005, 2006; Steeves et al., 2006; Busigny et al., 2010). Second, according to the divided labor account, the RH should still be more adept at processing Mooney faces than the LH, even if the former recruits the latter. However, the present results show that Mooney face processing in the RH was slower than in the LH. Finally, the divided labor theory does not provide any predictions or possible explanations for the opposing relations found in Experiment 2 between hemispheric processing of faces (natural and Mooney faces) and creative ability (both fluency and quality measures).

A few limitations exist in our study. First, in Experiment 1 we found a significant relationship between Mooney face processing and verbal, not visual, TACT scores. Aziz-Zadeh et al. (2013) also found in their visual creativity study activation in language-related brain areas. These authors interpreted their findings as related to the verbal component required in their visual tasks. Thus, the relation we find between Mooney face processing and TACT verbal scores may be explained by this interpretation as well. Second, in Experiment 2 we had significantly lower accuracy rates in the Mooney faces task compared to the natural faces and silhouette tasks. This low accuracy may be due to the high load demands imposed by the SVF paradigm that we used in Experiment 2. Third, our data collected in both experiments were skewed. Such skewedness can affect data analyses and interpretation. However, the analysis methods we used are generally robust against the assumption of normality (Schmider et al., 2010). Since using non-parametric methods of analysis lowers test power, we chose to remain with the standard methods we used. Finally, our fluency and quality measures of divergent thinking showed high correlations. These high correlations may be a result of fluency confound. It has been shown that the more responses a participant generates, the more these responses become unique (Beaty and Silvia, 2012). Thus, the participant’s quality scores may be confounded with their fluency of responses. Future research should use alternative methods to measure divergent thinking quality scores in relation to unconventional stimuli processing (Benedek et al., 2013; Silvia et al., 2013).

In conclusion, our results demonstrate that unconventional face processing correlates with aspects of creative ability. In addition, we show that while conventional faces are better processed by the specialized RH system, unconventional faces are better processed by the non-specialized LH system. Finally, only unconventional face processing by the non-specialized LH system was positively correlated with verbal and visual aspects of creative ability. Combined with previous findings in verbal creativity, a general hypothesis emerges. This hypothesis emphasizes the importance of the non-specialized lateralized system in creative processing. We suggest that creativity is a product of a dual system interaction in a given cognitive domain—a specialized system responsible for conventional processing and a non-specialized system responsible for unconventional processing. The interaction between these two systems allows for effective processing of both conventional and unconventional stimuli and may thus support creativity.

## Conflict of interest statement

The authors declare that the research was conducted in the absence of any commercial or financial relationships that could be construed as a potential conflict of interest.
